# Surgery of the Large Intestine

**Published:** 1902-02-08

**Authors:** 


### SURGERY OF THE LARGE INTESTINE.

At the last meeting of the Clinical Society, Mr. Arthur Barker showed two cases which illustrate well what can be done with the large intestine. The first was a case of excision of the cæcum in a man aged 30, who was operated on for acute obstruction in May, 1899. The enormously distended and hypertrophied small intestine was first incised and five pints or so of liquid fæces were evacuated. It could then be made out that the cause of the obstruction was a hard growth round the ilio-cæcal valve. The small gut was therefore anastomosed laterally, at a distance of about 2 feet from its termination, with the transverse colon. After six weeks a fresh incision was made over the cæcum which was excised, together with some inches of the ascending colon and ilium. A slight fæcal fistula remained for some weeks, but with this exception the recovery was perfect and the patient still remained in excellent health, being without any evidence of recurrence two years and eight months after the operation. The growth was a typical carcinoma. The second case was one of excision of the transverse colon in a woman aged 58 years who was operated on for carcinoma of the colon and chronic obstruction in October, 1900. At the time of operation she was, and had for long been, suffering from diabetes. Between 4½ and 5 inches of the transverse colon were removed and end-to-end anastomosis was effected. The dressings remained untouched for 10 days, but on their removal a small fæcal fistula was found to exist which discharged for some weeks. Otherwise recovery was uneventful. This growth also was typical carcinoma, and this patient also had been in first-rate health ever since the operation, and still showed no evidence of recurrence.

